# Costs and effects of the Tanzanian national voucher scheme for insecticide-treated nets

**DOI:** 10.1186/1475-2875-7-32

**Published:** 2008-02-15

**Authors:** Jo-Ann Mulligan, Joshua Yukich, Kara Hanson

**Affiliations:** 1 Health Economics and Financing Programme, Department of Public Health and Policy, London School of Hygiene and Tropical Medicine, Keppel Street, London, WC1E 7HT, UK; 2 Department of Public Health and Epidemiology, Swiss Tropical Institute, P.O. Box, 4002, Basel, Switzerland

## Abstract

**Background:**

The cost-effectiveness of insecticide-treated nets (ITNs) in reducing morbidity and mortality is well established. International focus has now moved on to how best to scale up coverage and what financing mechanisms might be used to achieve this. The approach in Tanzania has been to deliver a targeted subsidy for those most vulnerable to the effects of malaria while at the same time providing support to the development of the commercial ITN distribution system. In October 2004, with funds from the Global Fund to Fight AIDS Tuberculosis and Malaria, the government launched the Tanzania National Voucher Scheme (TNVS), a nationwide discounted voucher scheme for ITNs for pregnant women and their infants. This paper analyses the costs and effects of the scheme and compares it with other approaches to distribution.

**Methods:**

Economic costs were estimated using the ingredients approach whereby all resources required in the delivery of the intervention (including the user contribution) are quantified and valued. Effects were measured in terms of number of vouchers used (and therefore nets delivered) and treated nets years. Estimates were also made for the cost per malaria case and death averted.

**Results and Conclusion:**

The total financial cost of the programme represents around 5% of the Ministry of Health's total budget. The average economic cost of delivering an ITN using the voucher scheme, including the user contribution, was $7.57. The cost-effectiveness results are within the benchmarks set by other malaria prevention studies. The Government of Tanzania's approach to scaling up ITNs uses both the public and private sectors in order to achieve and sustain the level of coverage required to meet the Abuja targets. The results presented here suggest that the TNVS is a cost-effective strategy for delivering subsidized ITNs to targeted vulnerable groups.

## Background

The cost-effectiveness of insecticide-treated nets (ITNs) in reducing morbidity and mortality is now well established [1,2]. It is considered to be one of the most cost-effective ways of reducing the burden of malaria with an estimated cost per Disability Adjusted Life Year (DALY) averted of between $19 and $85 (1995 prices) [3]. International focus has now moved on how best to deliver ITNs to achieve a high level of coverage and what financing mechanisms might be used to achieve this. Recent debates have centred on the trade-off between the need for immediate impact and the long-term sustainability of increased coverage. Proponents of free distribution emphasize the urgency for immediate results, whereas those who favour a more pluralistic approach, including the development of domestic markets for ITNs, are keen to ensure the long term sustainability of delivery of ITNs [4-7].

The WHO Position Statement on ITNs recommends implementation of strategies to sustain high levels of long-lasting insecticidal net (LLIN) coverage ("keep up" strategies) in parallel with strategies for achieving rapid scale- up ("catch up" strategies) with an overall aim of achieving full LLIN coverage [8]. However, the statement also recognizes that commercial markets are a valuable source of nets and recommends that where strong commercial markets exist or are developing they should be encouraged. The benefits of this are identified as ensuring longer-term access to nets and enhancing management of logistics and education efforts. The statement argues that separating the delivery of a targeted subsidy and the ITNs through distribution of vouchers or coupons to a target population makes it possible to stimulate local trade by building and maintaining a countrywide network of outlets. In this way "commercial demand and the commercial market are strengthened while the burden on the public health system of the logistics and distribution of ITNs, including long lasting nets, and of the associated management functions, is reduced" [8]. Importantly, the WHO statement recognizes that a decision to use vouchers should be considered in light of local experience.

The policy in Tanzania has been to combine the approaches of a targeted subsidy for those most vulnerable to the effects of malaria while at the same time providing support to the development of the commercial ITN distribution system using a social marketing programme [9]. Social marketing in this context refers to a range of activities including improving the impact of locally manufactured nets by bundling them with long lasting insecticide and improving availability at the retail level. In 2008, this "keep up" approach will be reinforced by a massive "catch up" campaign for children aged between one and five who are not beneficiaries of the voucher programme.

In October 2004, with funds from the Global Fund to fight AIDS, Tuberculosis and Malaria (GFATM), the government launched the Tanzania National Voucher Scheme (TNVS), a nationwide discounted voucher scheme for ITNs for pregnant women and their subsequently new born infants. Table 1 shows the key events in the development of the scheme. Under the scheme, every pregnant woman who attends an antenatal clinic (ANC) is eligible to receive a voucher which can be used as part-payment for an ITN (defined as a conventional net bundled with a package of insecticide). The vouchers carry a fixed value: this was set at Tsh 2750 (around US$ 2) from October 2004 to December 2006, and was raised to Tsh 3250 from January 2007. Upon production of the voucher and her ANC card a woman can purchase any size of net using her voucher. This means that the value of the top-up amount is variable but in 2006 was around Tsh 1019 around 20% of the cost of a standard 4 × 6 net.

**Table 1:** Time line of key events

**Time**	**Activity**
Mar 2002	Contract signed with GFATM
Nov 2002	Agreement signed between MoH and GFATM
Mar 2003	GFATM Funds arrive
Apr 2003	ITN cell leader appointed with funds and technical assistance from Swiss Development Corporation and Swiss Tropical Institute
Oct 2003	Programme Assistant and advisor appointed
May 2004	Tender for contractors issued
Jun 2004	Contract issued to logistics and training contractors
Jul 2004	Roll out planning begins
Sep 2004	Rollout training begins Regional training begins for voucher redemption
Oct 2004	Contracts issued to auditors and monitoring and evaluation contractors
Oct 2004	TNVS scheme formally launched Voucher distribution begins phased roll-out by district
May 2006	100% nationwide coverage achieved

The TNVS was expanded from October 2006 onwards with funding from the President's Malaria Initiative (PMI) to provide a further voucher to the mothers and caretakers of infants aged nine months issued at the time of the measles vaccination, in order to provide continued protection for the child when it sleeps alone or with other siblings.

**Figure 1:**
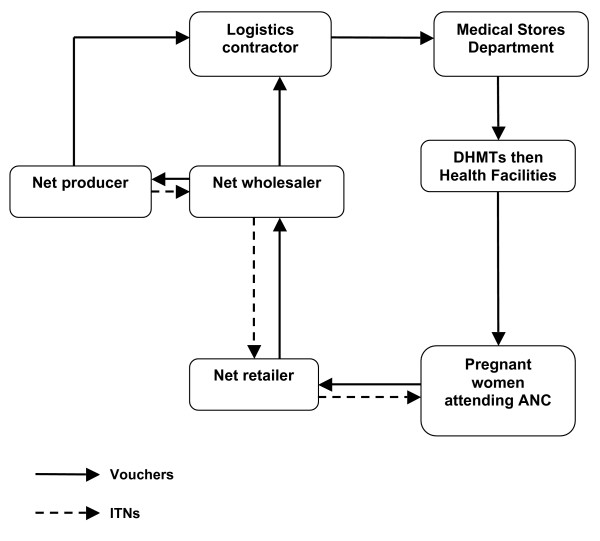
Tanzania National Voucher Scheme.

Implementation of the TNVS is through a public private partnership between the National Malaria Control Programme (NMCP), the district Council Health Management Teams (CHMTs), Reproductive and Child Health (RCH) facilities, private net manufacturers, over two hundred wholesalers, more than six thousand retailers and three non-governmental organisations (NGOs) contracted to the Ministry of Health. RCH staff and CHMTs are trained by World Vision Tanzania staff under a contract with the Ministry of Health and Social Welfare. Responsibility for voucher supply, distribution and redemption lies with the logistics contractor, Mennonite Economic Development Associates (MEDA), which procures vouchers and delivers them to district level. District Medical Officers (DMOs) are responsible for delivering vouchers to RCH facilities. RCH facilities distribute them to pregnant women who then redeem the vouchers for ITNs at local retailers (Figure 1). Redeemed vouchers are returned to wholesalers and then to manufacturers in exchange for new stock. Cash is provided against vouchers only at the very top of the system, to any of the four local manufacturers or a limited number of large wholesalers. This is to minimize the misuse of vouchers for products other than ITNs. A parallel system is used to supply free insecticide re-treatment kits to children attending vaccination clinics at three months and nine months, to encourage regular re-treatment of nets. The Medical Stores Department (an autonomous agency of the Ministry of Health) supplies insecticide re-treatment kits (IRK) directly to the districts through the distribution channels used for drugs and other medical supplies. A phased roll-out of the TNVS was launched in the first districts in October 2004, and all districts on the mainland were covered by May 2006.

The Ifakara Health Research and Development Centre and the London School of Hygiene and Tropical Medicine were contracted to undertake the monitoring and evaluation of the TNVS. The aim of this paper is to analyse the costs and effects of the scheme and to compare with other approaches to distribution. The perspective of the analysis is of both the provider and users whereby all costs and consequences attributable to the donors, the MOH and users were estimated.

## Methods

### Data collection

To ensure that this costing is comparable with future evaluations the analysis followed standardized guidelines recommended for costing ITN distribution systems [10]. Economic costs were estimated using the ingredients approach whereby all resources required in the delivery of an intervention are quantified and valued. This involved collecting actual line item expenditure and activity data wherever possible. In other instances, budget data were used to estimate expenditure. Project cost data were collected retrospectively from accounting records held by NMCP and the implementing partners. Data on the costs incurred by users were derived from the TNVS household survey and retail audit [11,12]. Household survey data also provided information on the mean number of children protected by a net bought with a voucher. Semi-structured interviews were held with key stakeholders and project staff to identify activities not recorded in project documentation and information on programme outputs. Estimates of ITN effectiveness (in terms of averted deaths and malaria cases) were derived from the literature [13].

### Categorization and analysis of costs

Economic costs or opportunity costs represent the value of resources in their next best alternative use. In this analysis economic costs differ from accounting financial costs in two main ways: first an equivalent annual capital cost is calculated for those items which are deemed to last more than one year and second any donated or subsidized goods and services (e.g. time of users to collect nets) are valued at their estimated market cost.

Cost identification was done by tracking the financial and economic costs associated with each activity. The time-frame for identifying costs ran from the first planning workshop held in early 2004 through to July 2006. The scheme involved four areas of activity: central management, training of key stakeholders, promotion activities and the logistics associated with the distribution, redemption and accounting for vouchers (see Table 2). Shared costs such as office equipment, office rent and office furniture and utilities were grouped as overheads. Budget estimates provided by the implementing partners were used to assign values to these items. Fixed asset registers were also used to identify and value capital equipment.

**Table 2:** Contributors to the intervention

**Contributor**	**Role**
Global Fund to Fight AIDS, Tuberculosis and Malaria	• Funder of TNVS main activities

Swiss Development Corporation (SDC) and Swiss Tropical Institute (STI)	• Funder of ITN cell

Ministry of Health National Malaria Control Programme (NMCP)	• Project management

Mennonite Economic Development Agency (MEDA)	• Logistics contractor • Voucher distribution and redemption

World Vision/CARE	• Conducting roll out training

Population Services International (PSI)	• Social marketing and technical support

District Health Management Team	• Costs of voucher distribution and reporting

Health Facilities	• Distribution of vouchers at ANC clinics

Retailers and wholesalers	• Handling vouchers

Households	• Top-up charges for ITNs • Time costs associated with collection of nets

Since 1999, a large network of stakeholders in Tanzania has promoted and supported a coordinated national ITN strategy (NATNETS). Household ownership of an ITN has increased substantially (from virtually nil to around 36% by 2007) and a strong commercial sector for the production, distribution and retailing of mosquito nets has emerged. The NATNETS strategy consists of four components: i) An ITN 'cell' based at the National Malaria Control Programme (NMCP), ii) SMARTNET a strategic social marketing program funded by the UK Department for International Development and the Royal Netherlands Embassy (this finished in June 2007), (iii) the provision of insecticide treatment and re-treatment kits to net manufacturers and retailers, and iv) the TNVS. A key decision in framing the analysis was determining the extent to which costs associated with the wider activities of the NATNETS programme should be included. While it is clear that the TNVS could not have taken place without the existence of wider social marketing activities, the focus of the present analysis are activities that are directly related to the voucher scheme only. The ITN cell of NMCP comprises a team leader and two programme staff. Following discussions with staff they are estimated to spend between 75 and 80% of their time on TNVS activities and this allocation was used in the baseline analysis. The activities of the wider NATNETS programme are the subject of a separate evaluation [14].

### Recurrent and capital costs

Expenditure was disaggregated by recurrent and capital expenditure, showing the difference between investment costs that are one-off and recurrent costs that are ongoing and represent the running costs of any programme implementation (see Table 3). Capital costs included formative research, building space, equipment for office use (e.g. computers), vehicles and ITNs (includes voucher subsidy and user contribution). Formative research refers to the costs of running a pilot voucher scheme in two districts but excludes on-going monitoring and evaluation activities. All capital items were annualized over assumed life spans using a discount rate of 3%. This enables an annual equivalent cost to be estimated that is then added to the annual recurrent estimate. This process reflects the value in use of capital items, rather than reflecting when the item was purchased. The useful life of vehicles was estimated at eight years. Formative research, computers, furniture and equipment were all estimated to have a useful life of five years. Nets were assumed to last for three years, although the effect of initial treatment and any subsequent re-treatments were assumed to provide one year of protection. Recurrent costs included staff related costs, consumables and fees (such as banking charges, service fees, and communication charges).

**Table 3:** Costs included in the analysis

	**Description of costs**
**Capital**	Formative research
	Planning costs
	Consensus building and meetings
	Initial training
	Buildings
	Vehicles
	Equipment and furniture
	ITNs (user contribution and subsidy)

**Recurrent**	Insecticide
	Personnel
	Fuel/Maintenance of vehicles
	Office/warehouse rental
	Advertising and promotion
	Supplies/overheads
	Management cost

All cost items were valued according to their market value in the year they were purchased in either Tanzanian shillings or US dollars, depending on where they were purchased. All costs were then translated into US dollars at the Tsh-US$ exchange rate for the year in which they were incurred (in July 2006 1340 Tsh = 1 US$). All costs are reported in 2006 US dollars.

An attempt was made to distinguish between start up costs and running costs of the programme. Start up costs are classified as one-time activities to get the programme up and running and includes formative research undertaken in two districts in 2004, planning activities by NMCP in the months leading up to the formal launch and the training of health workers by Care and World Vision. The start up costs of NMCP are defined as those relating to activities undertaken by the ITN cell staff from the appointment of the ITN cell leader to the official launch date in October 2004. In contrast to the formative research start up costs were not treated as a capital cost, as it is likely that some activities will need to be repeated at some point.

### Estimating user and health facility staff costs

The voucher scheme works on the basis that part of the price of the net is paid for by users. An important part of this analysis is the inclusion of user costs as well as the cost incurred by the providers of the voucher scheme. The retail audit and household survey provided information on the average top-up payment by users and typical travel costs involved in collecting nets [11,12]. The household survey reported that it takes women on average 40 minutes to get to a shop to redeem the voucher [11]. The opportunity cost of the total time taken to redeem the vouchers was based on the minimum wage of an agricultural worker and estimated to be Tsh 520 ($0.39). The associated average travel costs were found to be 68 Tsh ($0.05). Data from the retail audit indicated that the mean top-up price charged by retailers was Tsh 1019 ($0.76) [12]. This figure was varied in the sensitivity analysis.

The distribution of the vouchers at the health facility involves a number of activities by clinic staff including communal health education talks, explaining how the scheme works to individual women during antenatal clinic sessions, filling in the ledger books, collecting new voucher books and delivering used voucher books to the District Medical Officer (DMO). Although difficult to quantify it is important that these opportunity time costs are reflected in the results. Based on informal discussions with clinic staff and those involved in the monitoring and evaluation of the scheme it was estimated that these costs would amount to around five minutes per voucher distributed.

### Cost – effectiveness

As the effectiveness of ITNs in reducing infant and child mortality and improving maternal health has been amply demonstrated [13], no health impact data were collected as part of this study. The focus instead is on programme outputs i.e. the number of nets delivered to users. Only outcomes for children under five years of age are considered here. Estimating the number of malaria deaths averted by the intervention depends on net coverage, usage and the relationship between treated net years and mortality. The review by Lengeler found that in areas with stable malaria, ITNs reduced the incidence of uncomplicated malarial episodes by 50% compared to no nets [13]. They estimated that 5.5 lives could be saved each year for every 1,000 children protected with ITNs. In order to translate output data into health outcomes, the baseline analysis assumes that all vouchers redeemed at shops are used to purchase a net. Survey data from the TNVS household survey shows that on average each voucher net protects 0.88 children. This is reduced to 0.5 in the sensitivity analysis to take account of net wastage.

The outputs of the scheme are measured in terms of number of nets delivered to women and number of re-treatments performed. These outputs are combined into *treated net years (TNY)*. The combined indicator TNY is useful because it allows the inclusion of re-treatments of existing nets as part of the outcome measure. The WHO defines a conventionally treated net as any net that has been treated with a WHO recommended insecticide at least once a year [8]. The baseline analysis therefore assumes that either a re-treatment or a new ITN provides one year of protection for any individual using that net. The sensitivity analysis examines the impact of reducing insecticide effectiveness from 12 to 6 months. The following ratios are estimated: cost per voucher used (and, therefore, net delivered); cost per treated net year (TNY); cost per malaria case averted; cost per malaria death averted.

## Results

### Financial and economic costs

**Figure 2:**
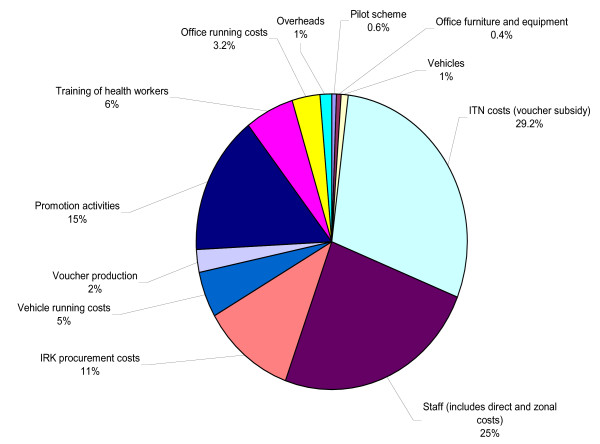
Breakdown of provider financial costs.

Over the first two years (2004–2006), the total provider financial costs of the TNVS programme were $10,680,516 (Table 4). Figure 2 provides a graphical breakdown of provider costs with staff costs making up the biggest component, followed by promotion activities and the costs of the ITNs themselves. Including the user contribution takes the total to $11,837,838. Economic costs include the user top up plus donated inputs in the form of ANC clinic time and user travel time. Capital costs are also annualized to reflect the annual equivalent value in use. The economic costs of the programme are $10,599,367 (see Table 5).

**Table 4:** Financial Costs, Provider plus User, 2004–2006

	**US$**	**%**	**Financial cost per voucher used (US$)**
**Capital costs**			
Formative research	68,736	1%	0.05
Office furniture and equipment	46,944	0%	0.03
Vehicles	83,110	1%	0.06
ITN costs (voucher subsidy)	3,123,294	29%	2.24
**Total Capital**	**3,322,084**	**31%**	
			
**Recurrent costs**			
Staff (includes direct and zonal costs)	2,609,508	24%	1.87
IRK procurement costs	1,200,355	11%	0.86
Vehicle running costs	522,204	5%	0.37
Voucher production	255,275	2%	0.18
Promotion activities	1,645,608	15%	1.18
Training of health workers	627,224	6%	0.45
Office running costs	347,077	3%	0.25
Overheads	151,182	1%	0.11
**Total recurrent**	**7,358,432**	**69%**	
**Total provider costs**	**10,680,516**		
			
**User direct costs**			
ITN user contribution	1,157,322	11%	0.83
			
**TOTAL FINANCIAL COSTS**	**11,837,838**		**8.49**

**Table 5:** Economic costs, 2004–06

	**$US**	**%**
**Capital**		
Formative research	27,121	0.3%
Office furniture and equipment	18,652	0.2%
Vehicles	21,394	0.2%
ITN costs (voucher subsidy)	2,153,047	20.4%
**Sub total**	**2,220,213**	**21.0%**
		
**Recurrent costs**		
Staff	2,609,508	24.7%
IRK procurement costs	1,200,355	11.4%
Vehicle running costs	522,204	4.9%
Voucher production	255,275	2.4%
Promotion activities	1,645,608	15.6%
Training of health workers	627,224	5.9%
Office running costs	347,077	3.3%
Overheads	151,182	1.4%
**Sub total**	**7,358,432**	**69.7%**
		
**User and ANC staff costs**		
ITN user contribution	797,802	7.6%
Direct travel costs	118,134	1.1%
ANC clinic staff costs	64,786	0.6%
**Sub total**	**980,722**	**9.3%**
		
**Total**	**10,559,367**	**100%**

### Cost per voucher used and per treated net year

As at July 31^st ^2006 2,424,987 vouchers had been sent to District Medical Offices for distribution to pregnant women. Of these 1,157,885 vouchers had been utilized by pregnant women and the vouchers returned to MEDA. Based on data from the household survey and from MEDA's own calculations from vouchers returned compared with matching stubs (counterfoils), NMCP estimates that returned vouchers represent around 83% of all vouchers actually utilized by women at that point. Therefore the actual number of voucher nets delivered to pregnant women using the TNVS was at least 1,395,042 at the end of July 2006. This figure represents the baseline output figure for all the cost-effectiveness estimates. On this basis the financial cost (including user top up) per voucher used, and therefore ITN delivered, is $8.49 and the economic cost is $7.57. A total of 1,600,000 insecticide re-treatment kits were also distributed by the programme during the same time period. The household survey found that 69% of mothers who received a re-treatment kit actually used it to retreat their nets and the baseline analysis is adjusted on this basis. If the impact of delivered voucher nets and re-treatment kits (adjusted for lower retreatment rates) are added together it is calculated this led to a total of 2,499,042 treated net years and a cost per treated net year of $4.23.

It is estimated that start up costs comprise around 8% of the total costs (see Table 6). If these costs are removed the economic cost per ITN delivered is estimated to be $6.93. The cost per treated net year is $3.87. The majority of start up costs represents training activities, some of which will need to be repeated at a future point in the scheme to ensure that skills and knowledge of health workers are maintained.

**Table 6:** Economic costs – 'start up' versus ongoing costs

	**US$**	**%**
**'Start up' costs**		
Formative research	27,121	0.3%
NMCP planning and pre launch activities	235,014	2%
Training of health workers	627,224	6%
**Sub total**	**889,359**	**8%**
		
**Capital costs**		
Office furniture and equipment	18,652	0%
Vehicles	21,394	0%
ITN costs (voucher subsidy)	2,153,047	20%
**Sub total**	**2,193,093**	**21%**
		
**Recurrent costs**		
Staff	2,374,494	22%
IRK procurement costs	1,200,355	11%
Vehicle running costs	522,204	5%
Voucher production	255,275	2%
Promotion activities	1,645,608	16%
Office running costs	347,077	3%
Overheads	151,182	1%
**Sub total**	**6,496,194**	**62%**
		
**User and ANC staff costs**		
ITN user contribution	797,802	8%
Direct travel costs	118,134	1%
ANC clinic staff costs	64,786	1%
**Sub total**	**980,722**	**9%**
		
**GRAND TOTAL**	**10,599,367**	**100%**
**TOTAL COST MINUS START UP**	**9,670,009**	

### Cost per malaria case and death averted

The programme resulted in a total of 2,499,042 treated net years and the mean number of children protected per voucher net is 0.88. If it is assumed that 1,000 treated ITNs will avert 5.5 child deaths per year [13], it is estimated that the programme averted 12,039 child deaths at an economic cost of $873 per child death averted. For averted malaria cases, the incidence of malaria outpatient attendances in children under 5 is reported as 723 per 1,000 per year (NMCP unpublished data). If it is assumed that ITNs lead to a 50% reduction in malaria incidence [13], it is expected that 794,995 malaria cases could be averted each year at a cost of $13 per malaria case averted.

### Sensitivity analysis

Sensitivity analysis was used to see if any of the results were sensitive to changes in uncertain parameters including: choice of discount rate; lifetime of vehicles; exchange rates; retail price of nets and proportion of nets retreated. On the effectiveness side the analysis varied the life time of ITNs, insecticide effectiveness, and utilization of ITNs amongst children. The main results from the sensitivity analysis are summarized in Table 7.

**Table 7:** Sensitivity analysis of selected parameters

**Variable**	**Baseline values**	**New value**	**Output indicators (baseline result)**			
			
			**Cost per voucher used ($7.57)**	**Cost per treated net year ($4.23)**	**Cost per malaria case averted ($13)**	**Cost per death averted ($873)**
User top up price	TZS 1019 ($0.83)	30% price reduction	$7.40	$4.13	$13	$857
		
		200% price increase	$8.14	$4.54	$14	$943

ITN Life expectancy	3 years	2 years	$8.59	$4.79	$15	$995

Mean number of children per net	0.88	0.5	$7.57	$4.23	$23	$1536

Insecticide re-treatment rates	69%	50%	$7.57	$4.81	$19.	$999

ITN definition	Treated within last 12 months	Treated within last 6 months	$7.57	$8.45	$27	$1754

Long lasting nets			$8.09	$2.70	$8.48	$560

On the cost side, changing the discount rate, useful life of vehicles, exchange rates and staff time administering the scheme had a negligible impact on overall economic costs and hence cost per ITN delivered. The average top up price charged was Tsh 1019 ($0.8). The sensitivity analysis examined the impact of increasing or lowering the top up amount by 30% and found that it had only limited impact on the cost per ITN delivered. However this assumes that the relationship between voucher redemption and price remains constant, which is unlikely. Examining the responsiveness (or price elasticity) of demand to changes in price is beyond the scope of this paper but will be the subject of future work using data from the household and retail surveys.

Varying parameters on operational and effectiveness variables had a bigger impact on results. The base case analysis assumes that 69% of re-treatment kits distributed are actually used to retreat nets. If this figure is reduced to 50%, the cost per treated net year increases from $4.23 to $4.81, the cost per case averted increases from $13 to $19 and the cost per death averted increases from $873 to $999. If the useful life of an ITN is assumed to be two rather than three years, the cost per ITN delivered increases to $8.59, the cost per treated net year is $4.79 and the cost per death averted is $995. If the mean number of children protected per voucher net falls from 0.88 to 0.5 the cost per malaria case averted increases from $13 to $23 and the cost per death averted increases from $873 to $1536.

Table 7 also shows the estimated economic cost per malaria case and death averted if all manufacturers switch to long lasting nets with three years life expectancy and a cost of $5. Aside from cost per ITN delivered, all cost per output ratios are substantially lower than with ordinary nets reflecting the greater effectiveness of long lasting nets versus the relatively high cost of procuring re-treatment kits and lower re-treatment rates associated with ordinary nets.

## Discussion and Conclusion

A total provider financial cost of $10.6 million for the delivery of 1.3 million voucher nets represents around 5% of the Ministry of Health's total budget. The average economic cost of the voucher scheme, including the user top-up, was found to be $7.57 per ITN delivered and $873 per death averted. An analysis of a free distribution of long lasting nets with a measles immunisation campaign in Togo reported a cost of $5.95 per net distributed, $4.40 per malaria case averted and $856 per death averted [15]. In Malawi, a study of social marketing programme which delivered heavily subsidized nets through health facilities reported a figure of approximately $3 per net delivered [16]. In line with other evaluations the costs presented here represent an economic cost [3,17]. Importantly, we have attempted to capture as fully as possible all costs associated with running a national scale ITN distribution programme, including all training and logistical activities. This analysis differs from many others in that it captures the full costs incurred including time and travel costs of users to collect the nets as well as the top up prices charged by retailers. While the results presented here are somewhat higher than that reported in Togo and Malawi they are well within the benchmarks set by other malaria prevention studies. A review of all ITN cost effectiveness studies for the Disease Control Priorities Project found that the cost per death averted varied from $254 to $3,437 [18,19]. The only other evaluation of a social marketing and voucher project, although on a smaller scale, reported a cost per death averted of $1,603 [20].

Given the absence of pre- and post-incidence data on malaria, the calculations of effectiveness presented here are based on national averages and are indicative only. These estimates rely on the assumption that coverage and utilization remain at the same rate over the two years. The estimate of ITNs effectiveness is conservative since it only included effects for children under five and did not include any beneficial effects on the mother or other family members. As the TNVS is a targeted programme it is expected that a high proportion of nets will be used to protect infants and varying the usage rate of ITNs among children has a strong impact on its cost-effectiveness.

This analysis did not estimate the wider effects of using the retail sector as a mechanism for delivering nets. Evidence from the TNVS retail survey indicates that nets are now very widely available even in rural areas [12]. It is not possible to judge at this stage the extent to which this is a result of the voucher scheme alone. However, together with the social marketing activities of SMARTNET, the existence of a public subsidy for ITNs is thought likely to draw more retailers to the ITN market as well encourage existing retailers to remain. This has the potential for wider economic benefits to wholesalers, retailers and ultimately consumers (in the form of lower prices) from an expanding market.

Cost-effectiveness analysis cannot address directly issues of sustainability or equity, but both are important to any discussion of how to scale up coverage, especially to the most vulnerable groups. A voucher system in the context of wider social marketing activities can promote sustainability objectives by providing a long-term system for distributing nets. But charging top up prices threatens equity objectives if the poorest groups cannot afford to pay. Delivery systems for ITNs have been debated for several years, but the evidence base on cost-effectiveness of alternative delivery systems is still largely drawn from small scale projects and relatively short follow-up periods, and does not yet reflect what would happen in large scale national programmes over time. More information is also needed on how to use existing market channels to distribute nets, free or otherwise. Continued monitoring and evaluation has a role to play in establishing the extent to which these distribution channels are sustainable and how they can supplement campaigns. Webster and colleagues note that free distribution or delivery of ITNs through integration with other campaigns (eg immunization) provides a fast catch-up solution to scaling up coverage [21]. But where no other system is in place to keep-up this coverage, ownership is transient. The TNVS is designed to keep-up coverage, but to properly compare the effectiveness and cost-effectiveness of these different systems, they should be monitored over a period of at least three to five years [21].

No-one can predict how the donor environment will change over the next five years, and there is no guarantee of long term commitment by donors for free net distribution. Even if that commitment is forthcoming, many believe exploring a mix of strategies of delivery which take account of the local context provides the best basis for scaling up [4,16]. In Tanzania it is recognized that neither the public sector nor the commercial sector alone can achieve and sustain the level of coverage required to meet the Abuja targets. Despite the success of free distribution systems for achieving rapid scale up of coverage, the WHO recognizes the importance of commercial channels in some settings [8]. Thus in Tanzania there has been a mixed approach to delivering ITNs: combining social marketing techniques with the targeting of vulnerable groups with subsidies [9]. The results presented here suggest that the TNVS, a key part of that strategy, is a cost-effective method of delivering subsidized ITNs to targeted groups.

## Authors' contributions

KH is a principal investigator for the overall monitoring and evaluation of the TNVS. All the authors contributed to the design of the study. JM and JY designed the data collection instruments. JM drafted the paper. All the authors critically revised the paper and approved the final manuscript.

## References

[B1] Wiseman V, Hawley W, Kuile F, Phillips-Howard P, Vulule J, Nahlen B, Mills A (2003). The cost-effectiveness of permethrin-treated bednets in an area of intense malaria transmission in western Kenya. Am J Trop Med Hyg.

[B2] Picard J, Aikins M, Alonso P, Armstrong Schellenberg J, Greenwood B, Mills A (1993). A malaria control trial using insecticide-treated bed nets and targeted chemprophylaxis in a rural area of The Gambia, West Africa. 8. Cost-effectiveness of bed net impregnation alone or combined with chemoprophylaxis in preventing mortality and morbidity from malaria in Gambian children.. Trans R Soc Trop Med Hyg.

[B3] Goodman C, Coleman P, Mills A (1999). Cost-effectiveness of malaria control in sub-Saharan Africa. The Lancet.

[B4] Muller O, Jahn A (2003). Expanding insecticide-treated mosquito net coverage in Africa: tradeoffs between public and commercial strategies. Tropical Medicine International Health.

[B5] Stevens W (2005). Untangling the debate surrounding strategies for achieving sustainable high coverage of insecticide treated nets. Applied Health Economics and Policy.

[B6] Curtis C, Maxwell C, Lemnge M, Kilama W, Steketee R, Hawley W, Bergevin Y, Campbell C, Sachs J, Teklehaimanot A, Ochola A, Guyatt H, Snow R (2003). Scaling-up coverage with insecticide treated nets against malaria in Africa: who should pay?. Lancet Infect Dis.

[B7] Lines J, Lengeler C, Cham K, de Savigny D, Chimumbwa J, Langi P, Carroll D, Mills A, Hanson K, Webster J, Lynch M, Addington W, Hill J, Rowland M, Worral E, MacDonald M, Kilian A (2003). Scaling-up and sustaining insecticide-treated net coverage. Lancet Infect Dis.

[B8] WHO (2007). Insecticide-Treated Mosquito Nets: a WHO Position Statement.

[B9] Magesa S, Lengeler C, deSavigny D, Miller J, Njau R, Kramer K, Kitua A, Mwita A (2005). Creating an "enabling environment" for taking insecticide treated nets to national scale: the Tanzanian experience. Malar J.

[B10] Kolaczinski J, Hanson K (2006). Costing the distribution of insecticide-treated nets: a review of cost and cost-effectiveness studies to provide guidance on standardization of costing methodology. Malar J.

[B11] Hanson K, Marchant T, Mponda H, Nathan R, Bruce J (2006). Report on 2006 TNVS household, facility services and facility users surveys.

[B12] Stephen G, Mulligan J, Ebenezeri S, Mtawa E, Hanson K (2006). Tanzanian National Voucher Scheme: Report on first round of retail census.

[B13] Lengeler C (2004). Insecticide-treated bed nets and curtains for preventing malaria. Cochrane Database Systematic Review.

[B14] Yukich J, Mulligan J, Hanson K, Brown N, Chavasse D, Stevens W, Justino J, Mtema J, Zerom M, Ghebremeskel T, Khouma M, Urrutia JM, McGuire D, Tediosi F, deSavigny D, Lengeler C (2006). The Costs and Cost-Effectiveness of Insecticide Treated Bed Net Distribution Systems in Sub-Saharan Africa: Atlanta, USA..

[B15] Mueller D, Wiseman V, Bakusa D, Morgah K, Dare A, Tchamdja P (2007). Economic evaluation of the Togo Integrated Child Health Campaign 2004.

[B16] Stevens W, Wiseman V, Ortiz J, Chavasse D (2005). The costs and effects of a nationwide insecticide-treated net programme: the case of Malawi. Malar J.

[B17] Hanson K, Goodman C (2004). The Economics of Malaria Control Interventions.

[B18] Breman J, Mills A, Snow R, Mulligan J, Lengeler C, Mendis K, Sharp B, Morel C, Marchesini P, White NJ, Steketee R, Doumbo O, Jamison D, Breman J, Measham A, Et al  (2006). Conquering Malaria. Disease Control Priorities in Developing Countries.

[B19] Mulligan J, Morel C, Mills A (2005). Cost-Effectiveness of Malaria Control Interventions. Disease Control Priorities Project Background Paper.

[B20] Hanson K, Kikumbih N, Armstrong Schellenberg J, Mponda H, Nathan R, Lake S, Mills A, Tanner M, Lengeler C (2003). Cost-effectiveness of social marketing of insecticide-treated nets for malaria control in the United Republic of Tanzania. Bull World Health Organ.

[B21] Webster J, Hill J, Lines J, Hanson K (2007). Delivery systems for insecticde treated and untreated mosquito nets in Africa: categorization and outcomes achieved. Health Policy Plan.

